# Investigation of the Effect of Formaldehyde on the Condition of Periodontal Tissues of Woodworking Industry Workers

**DOI:** 10.25122/jml-2020-0016

**Published:** 2020

**Authors:** Olha Mykhaylivna Tokar, Victor Markianovich Batig, Marianna Oleksandrivna Ostafiichuk, Mykola Olegovich Ishkov, Michael Ivanovich Sheremet

**Affiliations:** 1. Department of Therapeutic Dentistry, Bukovinian State Medical University, Chernivtsi, Ukraine; 2. Surgery Department No.1, Bukovinian State Medical University, Chernivtsi, Ukraine

**Keywords:** Formaldehyde, woodworking, periodontal tissues, periodontal screening test

## Abstract

The high prevalence of periodontal diseases in workers with professional contact with unfavorable factors of the production environment is an unresolved problem of dentistry. This study aimed to investigate the harmful effects of formaldehyde on periodontal tissues in woodworkers who have long-term contact with formaldehyde in their professional activities. Sixty-nine men with occupational exposure to formaldehyde were examined to study the effect of formaldehyde on the human periodontal tissues, looking particularly at signs of the periodontal tissues’ inflammatory process using a series of periodontal indices. The study results showed that the condition of periodontal tissues was statistically significantly worse in woodworkers who have long-term contact with formaldehyde in their professional activities. However, the hygiene status was not significantly different in the main group and the comparison group. Thus, we concluded that working under conditions of constant exposure to formaldehyde has a negative effect on the condition of periodontal tissues.

## Introduction

Studies on periodontitis stated that it is a chronic destructive disease characterized by inflammation of the supporting tissues of the teeth, resulting in periodontal tissue damage and alveolar bone loss [1-3].

Since periodontitis is one of the most prevalent diseases, several studies were conducted to investigate its pathogenesis [4-6].

The high prevalence of periodontal diseases in workers who have professional contact with unfavorable factors of the production environment is an unresolved problem of dentistry [7, 8]. Significant sensitivity of the structural components of the periodontium makes it vulnerable to the action of external physical and chemical stimuli, and the continuous and unnoticeable entry into the body of industrial xenobiotics creates chemical pressure, causes the emergence of immunosuppressive states, reducing specific and non-specific protection, violation of microbial equilibrium and reduction of bone mineral density [9, 10].

The woodworking industry belongs to the sector of industries with a high risk of occupational diseases [11, 12]. At a woodworking company, the main sources of pollution are drying shops, plywood manufacturing plants, boiler rooms, chipboard manufacturing plants, fiberboard, wood-based plastic, wood flour and chips, motor vehicles, and others. In the chipboard production process, urea-formaldehyde resins are commonly used to bond wood particles and laminates together [13, 14]. Both production and use of these bonding agents, as well as storing finished particle boards, may release formaldehyde in the atmosphere [15]. Many contemporary studies describe the emission of formaldehyde mainly from three sources: the residual formaldehyde present in the resin, which is used as adhesives for wood-based panels, formaldehyde formed by the polycondensation reaction between hydroxymethyl groups and formaldehyde released by hydrolytic degradation of hardened resin, especially under conditions of increased humidity and increased temperature [16]. Despite its wide range of applications, formaldehyde belongs to hazard class 2 and belongs to carcinogens [17]. That is why the concentration of the substance should be constantly monitored, especially in the work area. The total concentration limit is 0.5 mg/m^3^, the daily average is 0.003 mg/m^3^, and the maximum concentration is 0.035 mg/m^3^ [18]. Measurements of maximum permissible concentrations confirm that the concentrations of harmful substances sometimes exceed the permissible limits. For the most part, this is due to the imperfection of the cleaning equipment’s design, or the complete lack of filters in the ventilation systems, and the replacement of the more environmentally friendly methods of cleaning with cheaper ones.

Formaldehyde adversely affects the respiratory system, eyes, skin, genetic material, reproductive organs, and has a strong effect on the central nervous system. Well-known toxic effects of exposure to formaldehyde are irritation of mucous membranes and allergic sensitization of the skin. Despite numerous studies about the negative impact of formaldehyde on the body as a whole, on the skin, on the mucous membranes, its hypothetical involvement in the pathogenesis of periodontal diseases has not been sufficiently studied.

Therefore, the purpose of our study was to investigate the negative effects of formaldehyde on periodontal tissues in woodworkers who have long-term contact with formaldehyde in their professional activities.

## Materials and Methods

To study the negative effect of formaldehyde on the human periodontal tissues, 69 men from 21 to 49 years old with occupational exposure to formaldehyde were examined. For the comparison group, 69 volunteers of approximately the same age who have no contact with harmful factors of the production environment were examined. All surveyed were divided into three age groups: 21-30 years old (n=54), 31-40 years old (n=41), and older than 41 (n=43). The study did not include patients with severe dentition deformity, malocclusion abnormalities, diseases of the oral mucosa, as well as patients with exacerbations of somatic diseases.

The subject’s criteria were based on a questionnaire. It included standard demographic data (age), medical (guaranteed that all subjects had not been taking any medications or vaccinations, nor exposed to any radiation for one year before sample collection), lifestyle and vocational questions (working hours/day, years of exposure, precautionary measures taken, and others). The study selected the subjects who had worked for five years or more in the carpentry field.

We surveyed the employees according to existing guidelines, taking into account patient complaints, medical history, and oral examination. To determine the periodontal status of workers, we used a periodontal screening test (PSR test) and the papillary-marginal-alveolar index – PMA (according to Schour, Massler, in the modification of Parma). To determine the need for treatment of periodontal disease, the Community Periodontal Index of Treatment Needs (CPITN) index was determined. Numerous studies confirm the relationship between oral hygiene and the severity of pathological changes in periodontal tissues. Therefore, we used the Simplified Oral Hygiene Indices of Green J.C. and Vermillion J.K. to evaluate the state of oral hygiene.

Data were expressed as means ± standard deviations of the parameters evaluated. The Student paired t-test was used to compare intragroup and intergroup measurements. A level of significance of 0.05 was used for all statistical comparisons.

**Figure 1:**
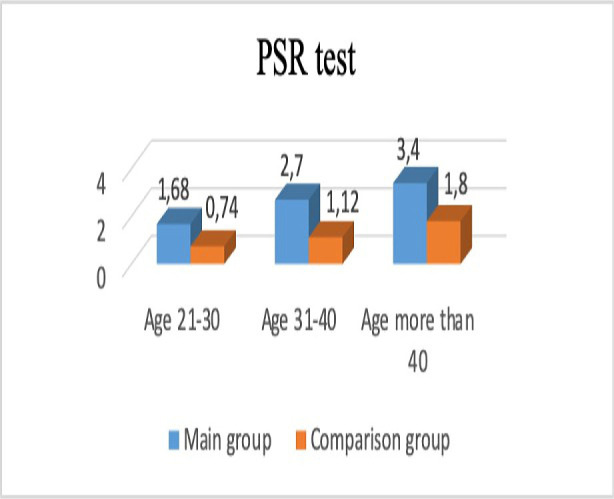
Results of the PSR test (р<0.05).

The results of the initial screening test of periodontal status (PSR test) of employees with long-term constant contact with paraformer dehydrate during their professional activities turned out to be significantly worse compared to the control group (Figure 1).

In patients aged 21-30 years old who have contact with paraformaldehyde during their professional activity, the average PSR test result was 1.68±0.12, which was significantly higher than the results of the same age group in the comparison group – 0.7±0.19.

In the second age group, the results of the PSR test in the main group significantly exceeded those in the comparison group (2.67±0.14 and 1.18±0.13, respectively). In the third age group, primary screening test scores were relatively high in both observation groups, but the main group’s results were also significantly higher compared to the comparison group (3.4±0.11 and 1.8±0.12, respectively).

We used the PMA index to determine the activity of the inflammatory process in periodontal tissues. The PMA index in people aged 21-30 years old of the main group was slightly higher than in people of the same age of the comparison group (Table 1). However, this difference is not statistically significant, which, in our opinion, is due to the relatively little work experience of this age group, and therefore not a significantly long period of contact with paraformaldehyde. On the contrary, indicators of this index were significantly different in the second and third age groups. Thus, it can be stated that with increasing age and, consequently, increasing work experience, the activity of inflammatory changes in periodontal tissues is higher in woodworking workers with lengthy contact with paraformaldehyde, than in people of the same age in the comparison group.

**Table 1:** Results of the PMA index.

**Observation groups**	**Age 21-30 (n=54)**	**Age 31-40 (n=41)**	**Age more than 40** **(n=43)**
**Main group**	0.38 ± 0.02	0.51 ± 0.04	0.59 ± 0.02
Comparison group	0.27 ± 0.05	0.29 ± 0.01	0.42 ± 0.03
The significance of the difference (p)	> 0.05	< 0.05	< 0.05

**Figure 2:**
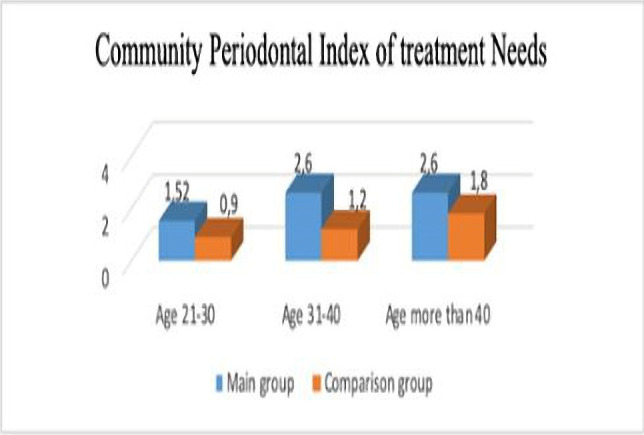
Results of the CPITN (р<0.05).

To determine the treatment need for periodontal tissue diseases, we used the Community Periodontal Index of Treatment Needs (CPITN) (Figure 2).

The value of the treatment need index was 1.52±0.08 in people of the main group that included subjects aged 21-30 years old, which was significantly higher than in the comparison group (0.9±0.2). In the second age group, the results of CPITN in the main group significantly exceeded those in the comparison group (2.65±0.13 and 1.2±0.08, respectively). In the third age group, CPITN scores were relatively high in both observation groups, but the results in the main group were also significantly higher than in the comparison group (2.6±0.11 and 1.8±0.11, respectively).

Numerous studies confirm the correlation between oral hygiene and periodontal tissue disorders. Therefore, one of the steps in our study was to evaluate the hygienic status of the oral cavity using the Simplified Oral Hygiene Index (OHI-S) (Table 2).

**Table 2:** Results of the OHI-S.

**Observation groups**	**Age 21-30**	**Age 31-40**	**Age more than 40**
**Main group**	0.84 ± 0.09	1.48 ± 0.09	1.73 ± 0.1
Comparison group	0.79 ± 0.16	1.34 ± 0.1	1.51 ± 0.1
The significance of the difference (p)	> 0.05	< 0.05	< 0.05

## Disscusion

We found that the oral health status in people of the main group and the comparison group (subjects aged 21-30 years old) is average, and the difference between them is not statistically significant. The same situation was observed in the 31-40 years age group; all subjects had an average level of oral hygiene with a not statistically significant difference. In the third age group (more than 40 years), in the main observation group, an unsatisfactory level of oral hygiene was found, while in the comparison group of the same age, an average level of oral hygiene was detected. However, the difference between the hygiene index of the main group and the comparison group was not statistically significant.

We surveyed 69 woodworkers who have long-term contact with paraformaldehyde in their professional activities. To compare the results, we surveyed 69 volunteers who had no contact with harmful environmental factors and came to the dental center for a prophylactic examination. The results of the initial screening of periodontal tissues showed that woodworkers who work under conditions of paraformaldehyde release have a statistically significantly worse condition of periodontal tissues than volunteers of approximately the same age who do not come into contact with harmful factors of the production environment.

Moreover, this difference between the PSR test increases with the age of the surveyed. So, the most significant difference between the index values was seen in the older age group (more than 40 years), which in our opinion is related to the increase of working experience in the wood industry and, therefore, more prolonged harmful effects of paraformaldehyde. The activity and prevalence of the inflammatory process in periodontal tissues according to the PMA index are statistically significantly higher in the woodworking industry workers in the middle and older age groups.

However, the difference between the values of this index of the main group and the comparison group at the age of 21-30 years is not statistically significant, which in our opinion is related to the small experience of the workers of this age. Determining the need for the treatment of periodontal tissue diseases showed the need for full periodontal treatment of most workers in the woodworking industry. Differences in the values of CPITN of the woodworking industry workers and the comparison group were statistically significant in all age groups. The state of oral hygiene of the woodworkers was slightly worse than the comparison group, but the difference was not statistically significant. Thus, in a similar oral hygiene condition in the woodworkers and the comparison group, we found a statistically significantly worse condition of periodontal tissues in the main group.

## Conclusions

After a detailed analysis of all indices, it can be stated that prolonged contact with paraformaldehyde in the occupational environment, in this case, the wood industry, leads to the occurrence or complication of preexisting periodontal tissue diseases. Moreover, increased contact with paraformaldehyde in the production environment due to an increase in work experience has a negative impact on the activity of the inflammatory process in periodontal tissues. The negative effect of paraformaldehyde on the state of periodontal tissues and the course of the inflammatory process in the gums in the woodworking industry workers cause the need to develop individual treatment and prevention of periodontal diseases in this population, which is a prospect of our further research.

## Conflict of Interest

The authors declare that there is no conflict of interest.
